# A health system approach to all-hazards disaster management: A systematic review

**DOI:** 10.1371/50081cad5861d

**Published:** 2012-08-22

**Authors:** Claire Bayntun

**Affiliations:** WHO Collaborating Centre for Mass Gatherings and Extreme Events, Health Protection Agency, London

## Abstract

AIM This review aims to develop disaster management practice using a health system strengthening approach through two objectives. Firstly, to review the disaster management literature to test the hypothesis that a holistic health system approach has not been established in practice or evaluated in the core literature. Secondly, to collate the worldwide experience of disaster management found in the core literature according to the components of a health system. METHOD A systematic review was conducted of the core literature published between January 2000 and November 2011 on the MEDLINE and Embase databases. Search terms combined the WHO’s descriptors for a health system with disaster terms. Non-restrictive inclusion criteria were applied. Papers were assessed using a quality appraisal tool. Content analysis identified the disaster management components discussed within the context of the health system. RESULTS The search yielded 143 relevant disaster management documents for collation. The review found that none of these publications described a holistic health system approach to disaster management, and none evaluated such an approach. CONCLUSION The findings of this review demonstrate that a holistic health system approach to disaster management has not been established in practice or evaluated in the core literature. Important lessons identified through the collation and analyses of isolated disaster-related experience require further research to incorporate them within a holistic health system approach. This approach, supported by the resolution passed at the World Health Assembly in 2011, aims to build health system resilience to protect immediate and long-term population health in the face of all-hazards disasters. Citation: Bayntun C. A health system approach to all-hazards disaster management: A systematic review. PLOS Currents Disasters. 2012 Aug 22. doi: 10.1371/50081cad5861d.

## Introduction

Disasters cause devastation for populations and can limit a health system’s progress for years ahead. In spite of funds being invested in disaster management in many countries, a comprehensive health system approach to build resilience in the face of disasters is still lacking. As a result, populations can suffer unnecessarily during and in the aftermath of disasters.

A resolution advocating a health system approach to disaster management was adopted at the World Health Assembly in May 2011, aiming to promote resilience and efficient recovery in the face of all-hazards disasters 1 . The health system can be defined as the structured and interrelated work of all agencies contributing to health within a country and “includes efforts to influence determinants of health as well as more direct health-improving activities” 2 . The WHO describes it as being composed of six building blocks - 1) service delivery, 2) health workers, 3) health information, 4) medical products, technology and vaccines, 5) health finance, and 6) governance and leadership 2 , which were originally derived from the World Health Report 2000 3 . These health system components will be described as ‘levers’ to better represent the dynamic, inter-dependent nature of the components.

WHO asks member states to prepare for ‘all-hazards’ disasters, whether natural or man-made. Historically, different aspects of disaster management have been considered and described in isolation. However, the ‘all-hazards’ approach to disaster management suggests that a health system needs resilience built into its structure across all levers. In this way, the health system can be strengthened to respond better to the challenges presented by any disaster.

The objective of this review is to use a health system approach to collate the worldwide experience of disasters, an initial step toward assessing the implementation of the World Health Assembly resolution of May 2011 1.

## Methods

This systematic review has been developed and written in light of the Preferred Reporting Items for Systematic Reviews and Meta-Analyses (PRISMA) Statement 4 . The Statement consists of a checklist intended to standardise the reporting of systematic reviews of randomised trials, and although it is not applicable in full to this review, it provides a useful tool to guide the structure and content of this report in accordance with the principles of transparent, comprehensive review reporting (Appendix 2).

**Protocol.** Information sources.Database searches were conducted using Embase and MEDLINE accessed via Ovid using ‘text words’ and ‘MeSH’ terms (Panel 1). In addition to the papers identified from these sources, a further 37 papers were selected for inclusion after being identified from the reference lists of retrieved articles and from citations.

Search limits. Papers included were those published in peer-reviewed core clinical journals between 1 January 2000 and 18 November 2011. Disaster management has been a rapidly evolving area over the last decade; thus this review concentrates on up-to-date information, while allowing the inclusion of materials describing a range of disasters across different continents. Following a pilot exploration of the literature, search terms were chosen to reflect the aim to identify a broad range of document type whilst avoiding the retrieval of an excessive number of irrelevant papers. Language restriction was not applied but all included papers were published in English. The search was limited to the ‘Human’ category. A second reviewer independently assessed the selection of documents according to the eligibility criteria. This resulted in two discrepancies which were considered further against objectives of the review, resulting in establishing a consensus for inclusion and exclusion.

**Panel 1 - Search terms, search strategy and results**

**Figure:**
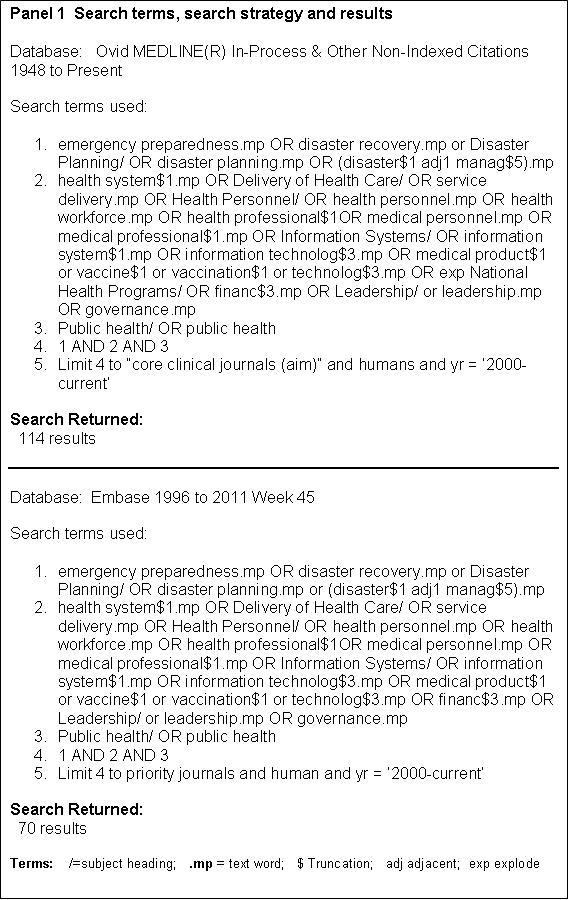

**Eligibility.** Retrieved records were excluded if the paper used the terms outside the context of disaster management or were of such a specific nature to warrant themselves inappropriate for the aims of the review (such as a new laboratory technique to identify a viral sub-strain).

Thus, inclusion criteria were broad, including reports of ‘round-table events’, evaluations of simulation exercises, opinion pieces and letters. Justification for the breadth of inclusion is that each document had value in reaching the objectives of the review – firstly to establish whether a health systems approach exists in the literature, and secondly to collate the worldwide experience of disaster management using a health system perspective in order to develop this approach. Figure 2 outlines the search and selection of papers.

**Flow diagram of the search and selection of papers:**
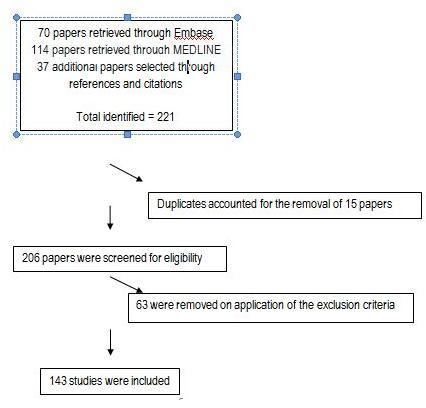

Data collection process. The initial piloting involved analysing 20 varied papers and confirmed the method of content analysis using a matrix combining the WHO’s six levers of health systems with components drawn from the WHO’s Global Assessment of National Health Sector Emergency Preparedness and Response^6^ 5 , the WHO’s baseline survey of member states (Matrix Template, Appendix 2).

A quality appraisal checklist was completed for each paper. Harden’s quality appraisal tool 6 was identified as the most suitable for public health literature (see Appendix 1). Appraisal details were recorded along with additional relevant comments outlining the relevance of each document (Summary Table of Reviewed Paper, Appendix 2).

## Results

The search strategy and application of the exclusion criteria yielded 143 relevant disaster management documents published between 1 January 2000 and 18 November 2011 (Figure 1). The review found that none of these publications described a holistic health system approach to disaster management, and none evaluated such an approach. A large proportion of the documents considered the importance of taking an all-hazards approach to disaster management, but described this through the context of isolated ‘vertical’ programmes, such as health professional training programmes or surge capacity development within hospitals.

Table 1 summarises how many papers address the WHO disaster management components 5 within each health system lever. While it should be expected that some areas are less well addressed by the literature, Table 1 shows that there is little coverage of complex and crucial issues such as international cooperation and partnership. Conversely, many publications highlight ‘vulnerabilities’ in disaster management and identify lessons for the ‘health sector plan’. The Summary Table of Reviewed Papers (Appendix 2) identifies which components are addressed in each publication. This enables valuable experience in building health system resilience to be located in the literature.

**Table 1 - Systematic review content analysis findings: Table shows the numbers of papers addressing disaster management components within the health system levers**

**Figure:**
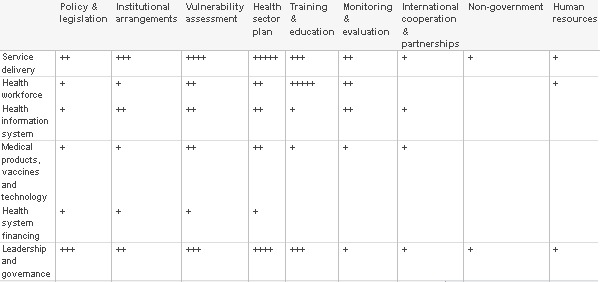

Key: Number of papers (range)

0 blank

1-10 +

11-20 ++

21-30 +++

31-40 ++++

41-50 +++++

## Conclusion

This review analyses and collates the worldwide experience of disasters found in the core literature within the context of health system levers. The aim and analysis structure of this review are innovative and the outcomes have international topical relevance and applicability, as evidenced by the recent World Health Assembly’s adopted resolution on this topic 1 and WHO Regional Office for Europe’s recent development of the WHO Toolkit for Evaluating Crisis Management 12.

This work provides an initial exploration of the evidence to support the aim that disaster management may be developed across countries using a health system approach. This paper calls for further research, or the publication of previously unpublished data, to establish objective measures to support policy and practice in integrating disaster management across the health system. This will contribute to sustainable health system resilience for populations in the face of all-hazards disaster.

## Discussion

The review has demonstrated that a holistic health system approach to disaster management has not been described in the core literature – not as primary research, wider articles, or as a topic for review (Panel 2). The WHO has placed an emphasis on health systems since the World Health Report in 2000, culminating in the 64th World Health Assembly in May 2011 adopting a resolution on ‘strengthening national health emergency and disaster management capacities and resilience of health systems’ 1 . This review establishes the current lack of data to support the implementation of the resolution. These collated results identify the valuable disaster management experience found in the core literature within the context of the health system levers. These findings can be used by researchers and practitioners to develop a holistic health system approach to disaster management, supporting the aims of the adopted World Health Assembly resolution.

**Panel 2 - RESEARCH IN CONTEXT: Previous reviews do not address the need for a holistic health system approach towards disaster management:** 

Further searches for relevant reviews were conducted on MEDLINE, Embase, PubMed, Database of Abstracts of Reviews of Effects (DARE) and The Cochrane Library databases. None of the searches identified papers describing the need for a holistic health system approach to developing disaster management practice. Other disaster-related reviews: • Savoia et al. reviewed the public health system research for emergency preparedness literature in the USA only. Their findings highlight the lack of any previous reviews in this area and the paucity of rigorous research in spite of the emergency preparedness literature having grown by about 33% per year since 2001 7. • Revere et al. reviewed 25 communication systems and tools for health-related emergency preparedness and response in the USA. They found this component of disaster management under-developed in the literature and the appropriate evaluation of the effectiveness of the systems absent 8. • Potter et al.’s review of the literature on emergency preparedness training effectiveness for the CDC was commissioned as a result of the increase in disaster management training opportunities but lack of a mechanism to objectively assess the effectiveness of training. They identify the need for evaluations of training, and that this should be based on performance improvement assessed through field exercises 9. • Auf der Heide highlights the importance of drawing on actual disaster experiences from the field to develop disaster management practice, identifying a series of disaster myths to demonstrate the impact of failing to incorporate learning from real disaster experiences 10. In summary, this review provides an important overview of disaster management from 2000 to 2011, collating evidence from the core literature at a time when this issue is receiving increased international attention. WHO member states and researchers are yet to adequately respond to the World Health Assembly’s 2011 resolution to build health system resilience to disasters 1. This review progresses that agenda; it identifies the lack of comprehensive disaster management implementation across health systems and the need for objective measures to be developed.

As a review of current experience, this work provides a platform for further qualitative analysis to identify the emerging themes and deliver practical, translatable evidence for multi-professional teams to build resilience across the health system in the face of all-hazards. For example, further qualitative analyses of these publications, not reported here, have been used to complement the WHO Toolkit on Evaluating Crisis Management for the WHO Regional Office of Europe 11 12. This recently developed tool aims to progress a health systems approach to disaster management^1^ 12.

**Strengths of this design and method.** The reviewed literature embodied a vast amount of information. A separate content matrix was originally completed on every paper reviewed, providing detail that can be used to guide disaster management from planning to acute response, recovery and rebuilding health systems post-disaster. The analysis and collation of this evidence within the context of health system levers would allow a holistic approach to disaster management to be progressed.

**Limitations.** The variety of papers analysed meant that some quality appraisal categories were not applicable for all documents; it was thus not appropriate to assign a numerical value to the quality appraisal. Qualitative comments were recorded for each paper to account for this issue. The Summary Table of Reviewed Papers (Appendix 2) presents comments that are pertinent to the aim of this review, including the type of evidence presented by the publications, such as ‘case report’. The use of peer-reviewed core journals from 2000 to 2011 establishes a quality standard on the otherwise diverse formats of the reviewed publications, while allowing the identified disaster management material to be up-to-date.

**Bias.** Bias is inherent to some of the individual documents reviewed due to the variety of formats included, such as opinion pieces and letters to journals. The qualitative nature of the documents means that this bias does not reduce the validity of each document for inclusion in the review, as they expose important areas of controversy and present pertinent, all be it often specific, experiences from the field. The nature of disasters means that most will not happen again in precisely the same way, so each experience and related lessons warrant consideration in their contribution to a health system strengthening approach to disaster management.

Overall, this review shows that much of disaster management research and commentary is taking place in the United States (USA), which is progressive in disaster management funding, theory, and institutions. Thus, a significant portion of the literature reviewed is USA-centric and reflects the availability of resources found only in the developed world. The disconnected nature of health disaster management in the USA means that much of the planning and resources have been implemented within isolation 13 , thus the literature generated is illuminating for all countries. This highlights the limitations and additional obstacles of not employing a health system wide approach.

A bias to the literature selection is that this review did not include documents from the grey literature. Instead, the objective was to establish whether the academic literature supported the objectives of the World Health Assembly resolution. I expect that inclusion of the grey literature would result in a less USA-centric view of disasters and management structures. An analysis of the wider relevant literature would be a worthwhile progression of this work.

**Implications for Public Health policy and practice.** The Summary Table of Reviewed Papers (Appendix 2) collates a diverse range of worldwide disaster experiences and identifies the specific literature which can provide valuable lessons. Professionals concerned with disaster management from around the world can use this review to identify developments to be made across all health system levers to enhance their preparedness and resilience to all-hazards disasters. This collation is important as much of the health system literature ignores the need to incorporate disaster planning and management.

This initial exploration provides scope for development. A holistic health system approach to all-hazards disaster management should reduce resource duplication at every phase of disaster management, maximise human and capital investment, optimise immediate and long term health outcomes, and prevent set-backs to a health system in the wake of a disaster 14 . Future health system research must address the issue of disaster management in response to the call made by this World Health Assembly resolution in May 2011. Evaluation methods need to be developed and conducted. Issues of practical implementation must be clarified so that priorities can be established, such as how to incorporate disaster management across local, national and regional levels to produce an integrated health system. Panel 3 provides a summary of how this paper aims to progress policy and practice.

**Panel 3 - Key Messages:** 

• The World Health Assembly adopted a resolution on this issue in 2011 1 with the aim to promote a health systems approach to disaster management to build resilience in the face of all-hazards disasters. • This systematic review of the core literature shows that a holistic health system approach to disaster management has not yet been adopted and there are no evaluations of the approach. • It provides a collation of the worldwide experience of disaster management as described in the core literature in the period 2000 to 2011. This is an initial step to progress research and practice in this area, describing the importance of a health systems approach to disaster management, and providing evidence to be used in developing policy and practice.

## Competing interests

The author has declared that no competing interests exist.

## Appendix A

### Quality appraisal criteria (Harden et al., 2004)

- An explicit theoretical framework and/or literature review
- Aims and objectives clearly stated
- A clear description of context
- A clear description of the sample and how it was recruited  (may not be relevant)
- A clear description of methods used to collect and analyse data/(information).
- Attempts made to establish the reliability or validity of data/(information) analysis
-  Inclusion of sufficient original data/(primary source information) to mediate between evidence and interpretation
- Other comments

## Appendix B

### PRISMA checklist, Matrix template and Summary table of reviewed articles

Appendices including the PRISMA checklist, the Matrix template and the Summary table of the reviewed articles are available here:

http://figshare.com/articles/Appendices_-_A_health_system_approach_to_all-hazards_disaster_management:_A_systematic_review/93444
